# Pancreatitis Associated with Viral Hepatitis: Systematic Review

**DOI:** 10.3390/jcm9103309

**Published:** 2020-10-15

**Authors:** Nikola Panic, Sladjana Mihajlovic, Miroslav Vujasinovic, Milutin Bulajic, Johannes-Matthias Löhr

**Affiliations:** 1 Faculty of Medicine, University of Belgrade, 11000 Belgrade, Serbia; nikola.panicmail@gmail.com (N.P.); drsladjanamihajlovic@gmail.com (S.M.); 2 Digestive Endoscopy Unit, University Clinic “Dr Dragisa Misovic-Dedinje”, 11000 Belgrade, Serbia; 3 Department for Digestive Diseases, Karolinska University Hospital, 14186 Stockholm, Sweden; miroslav.vujasinovic@sll.se; 4 Hospital Mater Olbia, 07026 Olbia, Italy; bulajic.milutin@gmail.com; 5 CLINTEC, Karolinska Institutet, 14186 Stockholm, Sweden

**Keywords:** pancreatitis, viral, hepatitis

## Abstract

Background: We conducted a systematic review in order to summarize the available data on pancreatitis associated with viral hepatitis. Methods: A comprehensive literature search of Medline, Scopus and ISI Web of Science databases was conducted and papers eligible for the inclusion identified. Results: In total, 46 studies reporting data on 73 patients were included in the analysis. Most of the cases were diagnosed in Asia (57.53%), followed by North America (23.29%), and Europe (13.70%). Most of the patients were affected by hepatitis A virus (HAV) (42.47%), followed by hepatitis E virus (HEV) (28.77%), hepatitis B virus (HBV) (8.22%), and hepatitis C virus (HCV) (1.37%), while 17.81% at the time of diagnosis were classified as affected by “hepatitis virus”. Pancreatitis was severe in 32.88% of cases. The respiratory system was affected in 2.74% of patients, 6.85% experienced renal failure, while 5.48% experienced a multiorgan dysfunction syndrome (MODS). Four patients (5.48%) needed pancreatic surgery. Despite the treatment, 21.92% of patients died. We identified fulminant hepatitis (*p* < 0.0001), MODS (*p* < 0.0001) and severe pancreatitis (*p* < 0.0001) to be significantly more present in patients who died in comparison to cured ones. Conclusion: Increased awareness of pancreatic involvement in viral hepatitis is needed because it can have a substantial impact on therapeutic approaches and outcomes.

## 1. Introduction

Acute pancreatitis (AP) represents an inflammatory condition of the pancreas associated with significant healthcare costs, morbidity and mortality [1]. Although usually having a mild clinical course, AP can develop into a severe and deadly disease with mortality reaching up to 15% in patients with necrosis [2]. Two main etiological factors associated with AP are gallstones and alcohol [3]. Nevertheless, infectious agents, such as viral ones, have also been implicated in AP pathogenesis [4].

Many viruses, such as the rubella virus, cytomegalovirus, Epstein–Barr virus and varicella zoster virus may lead to AP, with the mumps virus being the most common viral cause of AP [4]. Nevertheless, hepatitis viruses have also been implicated in the pathogenesis of AP. Hepatitis E virus (HEV) [5] and hepatitis A virus (HAV) [6] have been most frequently associated with AP. However, some authors also reported AP to be associated with a hepatitis B virus (HBV) [7], or hepatitis C virus (HCV) [8] infection. The mechanism behind these associations has been hypothesized to include an immune response or direct cytotoxicity against the infected acinar cells [9]. Other authors proposed the edema of the ampulla of Vater with the obstruction of pancreatic juice flow [10] to be a possible mechanism of viral damage of the pancreas.

In cases of fulminant hepatitis associated with AP, the severity of hepatitis itself has been reported to have a decisive impact on the treatment outcome [11]. However, in cases of AP associated with non-fulminant hepatitis [5,6], AP can have a significant impact on morbidity and mortality. Therefore, it is of great importance to recognize pancreatic involvement in hepatitis virus infections in time in order to modify the treatment accordingly and prevent the negative impact on outcome.

We conducted a systematic review in order to summarize the currently available data on pancreatitis associated with viral hepatitis.

## 2. Methods

We conducted and reported a systematic review following the PRISMA statement.

A literature search of Medline, Scopus and ISI Web of Science databases was conducted in order to identify papers reporting cases of pancreatitis associated with viral hepatitis. A combination of the following search terms was used (pancreas; pancreatic; pancreatitis) AND (viral hepatitis; hepatitis A; hepatitis B; hepatitis C; HBV; HCV; HEV; HAV).

The search was limited to papers published in English up to 1 July 2019. Additional papers were identified among references of previously retrieved studies as well as previously published reviews. The eligibility criteria for inclusion required that the study reported patient(s) affected by viral hepatitis and pancreatitis and that individual data on demographics, clinical presentation and outcomes were available. Viral hepatitis was defined as active hepatitis evoked by one of the hepatitis viruses (HAV, HBV, HCV, HEV).

Two investigators (NP and SM) independently extracted data. In case of any discrepancies regarding individual study inclusion, data extraction, or interpretation, a third investigator (JML) was consulted. The following data were extracted: first author name, year of publication, region of manuscript origin, patient gender, age at the diagnosis, presence of risk factors (alcohol, smoking, intravenous drug intake, comorbidities), clinical characteristics (symptoms and clinical presentation), diagnostics, treatment (pharmacological, surgical) and outcome (cured or died).

A descriptive analysis was conducted using proportion and mean ± SD when appropriate. A *t*-test, chi-square or Fisher’s test were used to compare data between the groups. Analyses were conducted using Stata software (StataCorp. 2019. Stata Statistical Software: Release 13. College Station, TX: StataCorp LP).

## 3. Results

**Figure 1:**
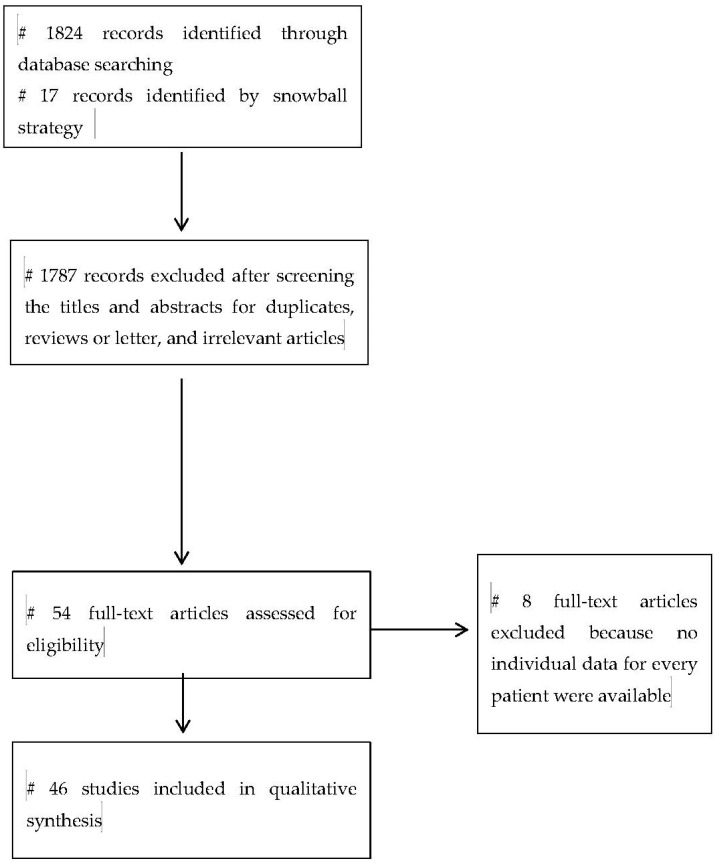
The search strategy and flow diagram for databases search.

Figure 1 depicts a flow chart of the search strategy and a diagram for paper selection (Figure 1).

In total, 54 case studies and case series reporting pancreatitis associated with viral hepatitis published from 1968 to 2019 were identified. However, 8 case studies and case series, in which individual data for each patient were not available, were excluded from the analyses. As a result, 46 studies reporting data on 73 patients were included [7,8,12,13,14,15,16,17,18,19,20,21,22,23,24,25,26,27,28,29,30,31,32,33,34,35,36,37,38,39,40,41,42,43,44,45,46,47,48,49,50,51,52,53,54,55].

Demographics and risk factors in 73 patients with viral hepatitis diagnosed with pancreatitis are reported in Table 1. The majority of patients were males (69.44%) diagnosed at a mean age of 24.47 ± 16.15. Most of the cases were diagnosed in Asia (57.53%, in India 53.52%), followed by North America (23.29), Europe (13.70%), South America (4.11%) and Africa (1.37%). Most of the patients were affected by HAV (42.47%), followed by HEV (28.77%), HBV (8.22%), and HCV (1.37%), while 17.81% of cases reported in older publications at the time of diagnosis were classified as affected by “hepatitis virus”. One case (1.37%) report referred to a case of pancreatitis that developed in the patient after combined HAV and HBV vaccination. Among potential risk factors, most frequently present was immunosuppression (5.48%) while alcohol and/or intravenous drug intake and chronic diseases were present in only a small portion.

Table 2 reports clinical characteristics of patients included in the analysis. Apart from jaundice (86.30%), the majority reported abdominal pain (82.19%) followed by fever (36.99%). In all the patients, pancreatic involvement initially presented itself in the form of acute pancreatitis, which in 32.88% of cases was severe. Necrotizing pancreatitis developed in 6 (8.21%) patients, pseudocysts in 4 (5.48%), while in 1 patient (1.37%), diagnosis of chronic pancreatitis was given during the follow-up. Hepatitis was fulminant in 17.81% of cases. Apart from the liver and pancreas, the respiratory system was affected in 2.74% of patients, 6.85% experienced renal failure, while 5.48% experienced a multiorgan dysfunction syndrome (MODS).

Table 3 reports the diagnostics that patients underwent. The majority of patients underwent abdominal ultrasound (69.86%), followed by computed tomography (CT) (61.64%), magnetic resonance cholangiopancreatography (MRCP) (4.11%), and endoscopic retrograde cholangiopancreatography (2.74%). All patients had features characteristic of pancreatitis upon abdominal imaging examination. In 10 (13.70%) patients, clinically relevant information on the disease was obtained in autopsy. Elevated lipase and amylase, when reported, were present in 100% and 98.33% of patients, respectively (when reported). The virus causing the infection was most frequently identified by serology (79.45%) and only in a minority of cases with polymerase chain reaction (PCR) (8.22%).

Table 4 reports data on treatment and outcome. In addition to being treated conservatively, 4 patients (5.48%) needed pancreatic surgery. Sixteen patients (21.92%) died during the course of the disease. When analyzing risk factors associated with deadly outcomes, we identified fulminant hepatitis (*p* < 0.0001) as well as MODS (*p* < 0.0001) and severe pancreatitis (*p* < 0.0001) to be significantly more present in patients who died in comparison to those who recovered.

## 4. Discussion

Our systematic review pooled the available data on demographics, clinical characteristics, diagnostics, therapies, and outcomes in patients with pancreatitis developed in settings of infection with hepatitis viruses.

The majority of the patients in our review were males in their third decade, coming from Asia (India). This is in line with the epidemiology of hepatitis viruses, as HAV and HEV, which caused the majority of pancreatitis cases in our review, are most frequently reported in Asia, particularly India [56]. Nevertheless, a substantial portion of the cases included in the review were diagnosed in North America and Europe, depicting the hepatitis burden in the Western world [57,58,59]. A typical patient in our review was not characterized by any well-known risk factors for the development of pancreatitis, such as alcoholism. However, a small portion was associated with immunosuppression of different causes (transplant patients, patients treated with steroids for other causes, patients affected by hematological diseases or cirrhosis), pointing towards the potential role of decreased immunity in making it possible for hepatitis viruses to affect the pancreas. The route by which the hepatitis virus can reach the pancreas has been hypothesized to be via blood or bile [8,29,60]. Nevertheless, no study so far has enlightened us as to the exact pathogenesis of pancreatitis associated with viral hepatitis. Possible mechanisms could include the development of edema of the ampulla of Vater with the obstruction of the outflow of pancreatic fluid [10]. Immune-modulating pathways resulting in a complex interaction of viral factors, such as genetics, with host factors including genetics and immunity, have recently been suggested to provoke extra-hepatic manifestations of HEV, including acute pancreatitis [61]. Further research is needed in order to reveal if pancreas damage during the infection with hepatitis viruses comes from viral replication itself or an immune-modulating mechanism such as molecular mimicry.

The relationship between viral hepatitis and AP may also be a result of pancreatic blood flow disorders due to infection with hepatitis viruses. A proper level of blood flow through the pancreas is a prerequisite for maintaining pancreatic integrity. Previous experimental studies have shown that disturbance of pancreatic blood flow may be the primary cause of AP [62]. Moreover, in the case of AP with a primary nonvascular mechanism of disease development, disturbances in pancreatic microcirculation quickly occur [63]. There is a strict correlation between the degree of pancreatic blood disturbance and the severity of AP [64]. On the other hand, improving pancreatic blood flow exhibits a protective effect in the pancreas [65,66]. Furthermore, clinical observations indicate that AP may develop as a result of circulatory disorders [67].

Patients presented jaundice, abdominal pain, and fever, which are all also symptoms of hepatitis, therefore being nonspecific for the involvement of the pancreas. However, almost all the patients in which amylase and/or lipase were measured showed significantly elevated levels. Furthermore, all patients subjected to imaging with US and/or CT demonstrated features associated with pancreatitis. This depicts that, with training, pancreatic involvement by hepatitis virus infection can be easily diagnosed.

Our review showed a significant death rate among patients with pancreatitis developed in the setting of a viral hepatic infection, being over 20%. This could be due to selection bias, as more serious cases of the disease tend to be recognized and published. Nevertheless, besides the severity of pancreatitis itself, we identified several important factors associated with death as an outcome. All but three of those patients who died developed pancreatitis associated with fulminant hepatitis, therefore confirming the previous reports of mortality depending largely on the severity of hepatitis, not pancreatitis [6,11]. Another association concerns the development of MODS, which exclusively developed in patients who did not survive, suggesting that affection of the pancreas can be a part of the generalized systemic response followed by multiorgan failure. In light of this, involvement of the pancreas in patients affected by viral hepatitis can be seen as one of the unfavorable prognostic signs, not only by causing a more severe clinical picture, but also pointing towards the potential involvement of other organs, leading to MODS.

We identified only one report of an associated infection with hepatitis viruses with the development of chronic pancreatitis [49]. Interestingly, that was the case of an infection with HBV. Only a minority of all the cases we included in our review referred to HBV or HCV infection [7,8,14,43,44,45,49]. However, as these are known to cause chronic hepatitis, it is reasonable to believe that, in the case of affection of the pancreas, chronic infection can also develop. Chronic pancreatitis caused by HBV or HCV can therefore be under-recognized and underreported. There is circumstantial evidence for this hypothesis, as both HBV and HCV chronic infections have been associated with increased risk for pancreatic cancer [68,69]. A plausible explanation for this association can be found in chronic inflammation of pancreas tissue caused by viral infection, and, in the case of HBV, being a DNA virus, in the integration of viral DNA in pancreatic tissue. Indeed, in situ hybridization and immunohistochemical techniques detected the HBsAg, serological markers of present or past HBV infection, in chronic inflammatory pancreatic acinar cells and in the pancreatic duct epithelia with pancreatic adenocarcinoma [70]. The HCV antigen was also found in pancreatic acinar cells [71]. Furthermore, another potential implication of chronic pancreatic involvement in viral hepatitis may concern development of pancreatic exocrine insufficiency (PEI) and/or diabetes mellitus. Indeed, HBV and HCV infections have been associated with type 2 diabetes [72,73], while no author associated hepatitis viruses with PEI. However, further research is needed in order to support these findings, especially as reports associating chronic HBV or HCV with chronic pancreatitis are lacking.

Our review has several limitations. Firstly, the studies included show great heterogeneity in the data available for extraction and subsequent synthesis. Heterogeneity was present not only between the included studies but also among individual cases within case series. Secondly, we identified a significant number of cases, published in languages other than English, that we could not include. However, we find this systematic review a valuable contribution to what is known on this subject, not only by summarizing the currently available evidence, but also by pointing out the direction needed for future research.

In conclusion, increased awareness of pancreatic involvement in viral hepatitis is needed, especially because it can have a substantial impact on therapeutic approaches and outcomes in affected patients.

## Figures and Tables

**Table 1:** Demographics and risk factors in 73 patients with viral hepatitis diagnosed with pancreatitis.

Age	24.47 ± 16.15
Age range	2–85
Gender	
Male	50 (69.44%)
Female	22 (30.56%)
Origin	
Asia	42 (57.53%)
Europe	10 (13.70%)
North America	17 (23.29%)
Africa	1 (1.37%)
South America	3 (4.11%)
Risk factors	
Immunosuppresion	4 (5.48%)
Alcohol	2 (2.74%)
Drugs	1 (1.37%)
Cirrhosis	1 (1.37%)
Pathogen	
Hepatitis A	31 (42.47%)
Hepatitis B	6 (8.22%)
Hepatitis C	1 (1.37%)
Hepatitis E	21 (28.77%)
Undefined	13 (17.81%)
Post-vaccination *	1 (1.37%)

* Pancreatitis developed post hepatitis A and B combined vaccine.

**Table 2:** Clinical characteristics of 73 patients with viral hepatitis diagnosed with pancreatitis.

Symptoms	
Fever	27 (36.99%)
Abdominal pain	60 (82.19%)
Jaundice	63 (86.30%)
Clinical presentation	
Acute pancreatitis	73 (100.0%)
Mild	48 (65.75%)
Moderate	1 (1.37%)
Severe	24 (32.88%)
Necrotizing pancreatitis	6 (8.21%)
Pseudocyst	4 (5.48%)
Chronic pancreatitis	1 (1.37%)
Extrapancreatic involvement	
FH	13 (17.81%)
Pulmonary (ARDS, pneumonitis)	2 (2.74%)
Renal failure	5 (6.85%)
MODS	4 (5.48%)

FH = fulminant hepatitis. ARDS = acute respiratory distress syndrome. MODS = multiple organ dysfunction syndrome.

**Table 3:** Diagnostics in 73 patients with viral hepatitis diagnosed with pancreatitis.

Imaging	
Abdominal ultrasonography	51 (69.86%)
Computed tomography	45 (61.64%)
Magnetic resonance cholangiopancreatography	3 (4.11%)
Endoscopic retrograde cholangiopancreatography	2 (2.74%)
Laparatomy	1 (1.37%)
Autopsy	10 (13.70%)
Laboratory	
Elevated lipase *	40 (100.00%)
Elevated amylase *	58 (98.33%)
Virus identification method	
Serology	58 (79.45%)
PCR	6 (8.22%)

PCR = polymerase chain reaction. * = percentage reported in relation to number of papers with data available.

**Table 4:** Treatment and outcome in 73 patients diagnosed with viral pancreatitis.

Therapy	
Pharmacological	69 (94.52%)
Surgical	4 (5.48%)
Follow up (months)	6.65 ± 8.92
Outcome	
Cured	57 (78.08%)
Died	16 (21.92%)
